# Comprehensive Evaluation of Gastrointestinal Factors Influencing the Stability and Function of mRNA‐Encapsulated Lipid Nanoparticles

**DOI:** 10.1002/ddr.70392

**Published:** 2026-09-23

**Authors:** Toma Shinkai, Koki Ogawa, Mohamed S. Mohamed, Tetsuya Ozeki

**Affiliations:** ^1^ Drug Delivery and Nano Pharmaceutics Graduate School of Pharmaceutical Sciences, Nagoya City University Nagoya Aichi Japan; ^2^ Department of Pharmaceutics and Pharmaceutical Technology, Faculty of Pharmacy Al‐Azhar University Assiut Branch Assiut Egypt

**Keywords:** lipid nanoparticle, mRNA, oral delivery

## Abstract

Owing to the intrinsic instability of mRNA, its routes of administration are limited, and to date, only injectable delivery has been clinically applied. In contrast, oral administration exposes mRNA to a variety of substances in the gastrointestinal (GI) tract. Despite these challenges, oral delivery remains the most familiar and convenient route for patients, making the development of orally administrable mRNA‐encapsulated lipid nanoparticles (mRNA–LNPs) highly desirable. However, no studies have directly examined how mRNA–LNPs are affected within the GI environment. In the present study, we investigated the effects of representative GI components on mRNA–LNPs. Among several digestive enzymes, pepsin markedly reduced the protein‐expression efficiency of the particles, whereas amylase had no effect. Pepsin‐treated particles exhibited increased size, suggesting that structural alterations contributed to the observed functional loss. Lipase at high concentrations impaired nanoparticle function. Because lipase hydrolyzes ester bonds in lipids, we examined LNPs incorporating C12‐200, an ionizable lipid lacking ester bonds, instead of SM‐102. The C12‐200 formulation showed reduced susceptibility to lipase‐induced functional loss, indicating that enzymatic lipid degradation underlay the observed effects. Simulated gastric fluid also significantly diminished particle functionality. A detailed analysis of the relationship between functionality and pH revealed that impairment primarily occurred at approximately pH 1 but not at pH ≥ 3. Collectively, these findings identify the key GI factors that compromise mRNA–LNP integrity and provide important insights into the rational design of orally administrable mRNA–LNP formulations.

## 1 Introduction

Therapeutics based on mRNA represent a versatile modality in which therapeutic effects are achieved through *in vivo* expression of encoded proteins. Because they can be applied to a broad spectrum of diseases, including infectious diseases, cancer, and genetic disorders, mRNA‐based medicines are emerging as promising next‐generation therapeutic platforms (Sahin et al. 2014). Owing to the intrinsic instability of mRNA under physiological conditions, clinical formulations rely on encapsulation within lipid nanoparticles (LNPs) (Tenchov et al. 2021). This approach enhances mRNA stability and its cellular uptake efficiency; therefore, LNPs are widely adopted because of their high encapsulation efficiency (EE) and robust delivery performance.

Clinically approved mRNA‐encapsulated LNP (mRNA–LNP) vaccines, such as those developed for COVID‐19, are intramuscularly administered (Ye et al. 2023). Despite their success, injectable administration has several drawbacks including invasiveness and challenges related to hygiene and infrastructure in resource‐limited settings. These limitations underscore the need to explore alternative delivery routes. Oral administration is non‐invasive, convenient, and generally preferred by patients, while avoiding issues associated with needle use. However, the gastrointestinal (GI) tract presents particularly harsh conditions for mRNA–LNPs, including drastic fluctuations in pH and exposure to various digestive enzymes (Hossian et al. 2019). Unlike intramuscularly administered particles, orally delivered LNPs must withstand these stresses, which may induce unpredictable structural and functional changes, potentially resulting in a substantial loss of activity. No detectable protein expression was observed following oral administration of conventional mRNA–LNPs to mice (Figure S1). This result suggests the limitations of current mRNA–LNP formulations for oral delivery.

Oral delivery of lipid‐based nanoparticles has been extensively studied. Bile acid‐incorporated liposomes coated with enteric polymers exhibit improved stability in the GI tract (Alghurabi et al. 2022). However, as liposomes substantially differ in structure from mRNA–LNPs, these strategies cannot be directly translated into mRNA–LNP formulations. Recently, the uptake of mRNA–LNPs has been enhanced by intestinal epithelial cells, which are a major barrier to the functionality of orally administered nanoparticles (Shinkai et al. 2024). Although this approach can potentially improve particle performance, it does not confer protection to particles within the GI tract. Studies have attempted to enhance the resistance of mRNA–LNPs to gastric or bile acids through compositional modifications (Luo et al. 2024; Suri et al. 2025); however, these approaches have targeted only specific and limited conditions. In realistic oral‐delivery scenarios, multiple digestive factors can simultaneously affect particle integrity, underscoring the need for a highly comprehensive understanding. However, detailed insights into specific substances that affect mRNA–LNPs remain limited. No study has thoroughly analyzed how the GI environment influences mRNA–LNPs during oral administration. Therefore, elucidating how individual factors within the GI tract affect the physicochemical properties and functional integrity of mRNA–LNPs may provide a rational and critical basis for formulation design aimed at enabling oral delivery.

In this study, we aimed to identify the specific environmental factors within the GI tract that influence the stability and functional performance of orally delivered mRNA–LNPs. We evaluated the physicochemical properties and protein‐expression efficiency of mRNA–LNPs after exposure to various solutions representative of the GI conditions to delineate the key factors that compromise particle stability or functionality for providing critical insights to guide rational development of strategies enabling oral administration of mRNA–LNPs.

## 2 Materials and Methods

### 2.1 Materials

SM‐102 and DSPE‐PEG‐NH_2_ were purchased from BroadPharm (San Diego, CA, USA). DSPC and DMG‐PEG 2000 were purchased from NOF (Tokyo, Japan). Cholesterol, C12‐200, pepsin (from Porcine Stomach Mucosa), lipase H (from *Candida cylindracea*), α‐amylase (from *Bacillus amyloliquefaciens*), and Triton X‐100 were purchased from Fujifilm Wako Pure Chemical Industries (Osaka, Japan); 1,1′‐dioctadecyl‐3,3,3′,3′‐tetramethylindodicarbocyanine 4‐chlorobenzenesulfonate salt (DiD) was purchased from Setareh Biotech (OR, USA). Luciferase Substrate (PicaGene Luminescence Kit) was purchased from TOYO B‐Net Co. Ltd. (Tokyo, Japan). Agarose S was purchased from NIPPON GENE (Tokyo, Japan). Sepharose CL‐4B was purchased from Merck (Darmstadt, Germany).

### 2.2 Preparation of mRNA–LNPs

Luciferase and ZsGreen1 mRNA were synthesized by standard *in vitro* transcription methods as previously described (Ogawa et al. 2024). mRNA–LNPs were prepared as previously described, with slight modifications (Ogawa et al. 2025). Briefly, a lipid mixture (1.45 mM) comprising SM‐102, ALC‐0315 or C12‐200 (ionizable lipid), DSPC, cholesterol, and DMG‐PEG 2000 at a molar ratio of 50:10:38.5:1.5 was prepared and mixed with the mRNA solution in a 50 mM citrate buffer (pH 3) at a lipid:mRNA volume ratio of 1:2.5 using a vortex mixer. For flow cytometry and fluorescence microscopy, DiD (0.5 mol% of total lipids) was incorporated into the lipid mixture. The resulting crude mRNA–LNPs were dialyzed against phosphate‐buffered saline (PBS, pH 7.4), concentrated by ultrafiltration using Amicon Ultra‐0.5 centrifugal filters (100 kDa MWCO; Merck Millipore, Burlington, MA, USA), and subsequently passed through a 0.45‐µm syringe filter. Particle size was measured using a Zetasizer Nano‐S (Malvern Instruments Limited, Malvern, UK).

Unless otherwise stated, all experiments were performed using mRNA–LNPs formulated with SM‐102. For selected experiments, mRNA–LNPs formulated with ALC‐0315 or C12‐200 were additionally prepared and evaluated.

### 2.3 Determination of EE

EE of each particle was determined by measuring the concentrations of total and free mRNA using a QuantiFluor RNA System (Promega Corporation, Madison, WI, USA). Total mRNA concentration was measured after solubilizing mRNA–LNPs with Triton X‐100 to release encapsulated mRNA. Free mRNA concentration was measured using intact mRNA–LNPs. EE was calculated using the following equation:

(1) $$\mathrm{EE}\;(\%)=\frac{\mathrm{mRNA}\;\left(\mathrm{total}\right)-\mathrm{mRNA}\;(\mathrm{free})}{\mathrm{mRNA}\;(\mathrm{total})}\times 100.$$

### 2.4 Treatment of mRNA–LNPs Using Test Solutions

To evaluate the effects of pH, cholic acid, and various enzymes on mRNA–LNPs, test solutions were prepared as summarized in Table 1. Each test solution was mixed with the mRNA–LNP suspension at a volume ratio of 9:1 and incubated at 37°C for 2 h. For the pH‐only study, PBS adjusted to pH 1, 3, 5, 7.4, or 9 was mixed with the mRNA–LNP suspension and incubated at ambient temperature (15°C–30°C) for 30 min. After incubation, pH of each suspension was neutralized using HCl or NaOH, and the resulting samples were characterized.

**Table 1:** Test solution.

Name	Composition
PBS (Control)	KH_2_PO_4_ (1.47 mM), KCl (2.68 mM), Na_2_HPO_4_ (8.10 mM), NaCl (137 mM)
SGF	HCl (84 mM), NaCl (34.2 mM), pH 1.2
SIF	KH_2_PO_4_ (50 mM), NaOH (23.6 mM), pH 6.8
Pepsin solution	SGF, Pepsin (0, 0.5, 1, 2, 3.2 mg/mL), pH 3 by NaOH
Cholic acid solution	SIF, Cholic acid (0, 5, 10, 15 mM), pH 6.8
Lipase solution	SIF, Lipase (0, 50, 100, 150 μg/mL), pH 6.8
Amylase solution	SIF, Amylase (0, 0.23, 0.46, 0.69 mg/mL), pH 6.8
PBS (acidic)	PBS (Control), pH 1, 3 or 5 by HCl
PBS (basic)	PBS (Control), pH 9 by NaOH

Abbreviations: PBS, phosphate‐buffered saline; SGF, simulated gastric fluid; SIF, simulated intestinal fluid.

### 2.5 Transmission Electron Microscopy (TEM)

The morphology of mRNA–LNPs after treatment with each test solution was examined by TEM. Following treatment, the particle suspensions were purified by ultrafiltration to remove any residual impurities. TEM was performed using a JEM‐1400Plus microscope (JEOL Ltd., Tokyo, Japan) after negative staining with 2% uranyl acetate.

### 2.6 Cell Culture

Caco‐2 and HEK293 cells (RIKEN BRC, Tsukuba, Japan) were cultured in Dulbecco's Modified Eagle Medium supplemented with 10% fetal bovine serum and 1% penicillin/streptomycin. Cells were maintained at 37°C in a humidified atmosphere containing 5% CO_2_. For differentiation, Caco‐2 cells were seeded at a density of 1 × 10^5^ cells/cm^2^ in 24‐well plates or 35 mm glass‐bottom dishes and cultured for 2 weeks, with the culture medium replaced every 2 days.

### 2.7 Luciferase Assay

Luciferase expression in Caco‐2 and HEK293 cells was measured to evaluate the efficiency of mRNA delivery. Each mRNA–LNP formulation (0.5 or 0.2 μg/mL mRNA) was added to differentiated Caco‐2 or HEK293 cells, respectively, cultured in 24‐well plates. Cells were harvested after 24 h of incubation, and proteins were extracted using a lysis buffer consisting of 0.1 M Tris, 2.5 mM ethylenediaminetetraacetic acid (EDTA), and 0.05% Triton X‐100 (pH 7.8). Luciferase activity in cell lysates was quantified using a Picagene luciferase substrate (TOYO B‐Net Co. Ltd.) and normalized with respect to total protein concentration.

### 2.8 Flow Cytometry

Intracellular uptake of mRNA–LNPs was quantified by flow cytometry. DiD‐labeled mRNA–LNPs (0.5 μg/mL mRNA) were added to differentiated Caco‐2 or HEK293 cells cultured in 24‐well plates. After 24 h of incubation, cells were trypsinized and analyzed using a FACSCanto II or FACSLyric flow cytometer (BD Biosciences, San Jose, CA, USA).

### 2.9 Confocal Laser Scanning Microscopy (CLSM)

Intracellular uptake of mRNA–LNPs was visualized by CLSM. DiD‐labeled mRNA–LNPs at an mRNA concentration of 1.25 μg/mL were added to differentiated Caco‐2 cells cultured in 35 mm glass‐bottom dishes. After 24 h of incubation, cells were stained with Hoechst 33342 to label the nuclei and were subsequently observed using a CLSM system (LSM800 with Airyscan; ZEISS, Jena, Germany).

### 2.10 Evaluation of mRNA Integrity in Gastric Conditions

To assess the effect of hydrogen ion concentration on mRNA integrity, simulated gastric fluid (SGF; pH 1.2 or 3) or PBS (pH 7.4) were used as test solutions. Each test solution was mixed with the mRNA suspension at a volume ratio of 9:1 and incubated at room temperature for 30 min. After incubation, the pH of each mixture was neutralized, and the samples were subjected to electrophoresis and gel permeation chromatography (GPC).

### 2.11 Electrophoresis

A 4 µL aliquot of a 50% (v/v) glycerol solution was added to 20 µL luciferase mRNA suspension (final concentration, 20 µg/mL) that had been treated with the test solution, and 20 µL of the resulting mixture was loaded onto a 1% (w/v) agarose gel. Electrophoresis was performed at 100 V for 20 min. The mRNA bands were visualized using a UV transilluminator (High‐Performance UV Transilluminator; Funakoshi Co. Ltd., Tokyo, Japan) and imaged using a smartphone camera.

For electrophoretic analysis of encapsulated mRNA, 10 µL of mRNA–LNP suspensions prepared with SM‐102 and treated with each test solution (final mRNA concentration, 50 µg/mL) was mixed with 10 µL of Triton X‐100 solution (0.8% (w/v) in TE buffer). Subsequently, 4 µL of a 50% (v/v) glycerol solution was added, and 20 µL of the resulting mixture was loaded onto the gel. The test solutions used were pepsin solutions (0 and 3.2 mg/mL), sodium cholate solutions (0 and 15 mM), lipase solutions (0 and 150 µg/mL), and amylase solutions (0 and 0.69 mg/mL). Electrophoresis was performed using a 1% (w/v) agarose gel at 50 V for 20 min. Naked mRNA (50 µg/mL) was also mixed with a 50% (v/v) glycerol solution, and 20 µL was loaded onto the gel.

### 2.12 GPC

GPC was performed using centrifuge columns (Pierce Centrifuge Columns, 2 mL; Thermo Fisher Scientific, Waltham, MA, USA) packed with Sepharose CL‐4B as the stationary phase, with Tris‐EDTA buffer used as the mobile phase. Luciferase mRNA treated with the test solution (4.75 µg mRNA) was applied to the column, and eluent fractions were collected at 2 min intervals. The amount of mRNA in each fraction was determined based on the mRNA concentration and corresponding fraction volume.

### 2.13 In Vivo Oral Administration of mRNA–LNPs

To evaluate the efficacy of mRNA–LNPs following oral administration in vivo, mRNA‐LNPs encapsulating firefly luciferase mRNA (CleanCap® FLuc mRNA, TriLink BioTechnologies, San Diego, CA, USA) and formulated with SM‐102 were orally administered to mice at a dose of 15 μg mRNA/mouse. fLuc expression was assessed 6 h after administration using an In Vivo Imaging System (IVIS; IVIS lumina Series III, Revvity, Waltham, MA, USA) after mice were administered with D‐Luciferin (4.5 mg/mouse).

### 2.14 Use of Artificial Intelligence (AI)‐Assisted Tools

An AI‐assisted language model (ChatGPT, OpenAI) was exclusively used for English language editing and stylistic refinement of the manuscript. This tool was not used to generate scientific content, interpret data, design experiments, or draw conclusions.

### 2.15 Statistical Analysis

The data are expressed as mean ± standard deviation. Statistical analyses were performed using EZR. Comparisons between two groups were conducted using student's *t*‐test. For comparisons among three or more groups, Tukey's multiple comparison test was used when no control group was included, whereas Dunnett's multiple comparison test was used when comparisons were made with a control group. A *p‐*value < 0.05 was considered statistically significant.

## 3 Results

### 3.1 Effect of SGF on the mRNA–LNPs

**Figure 1:**
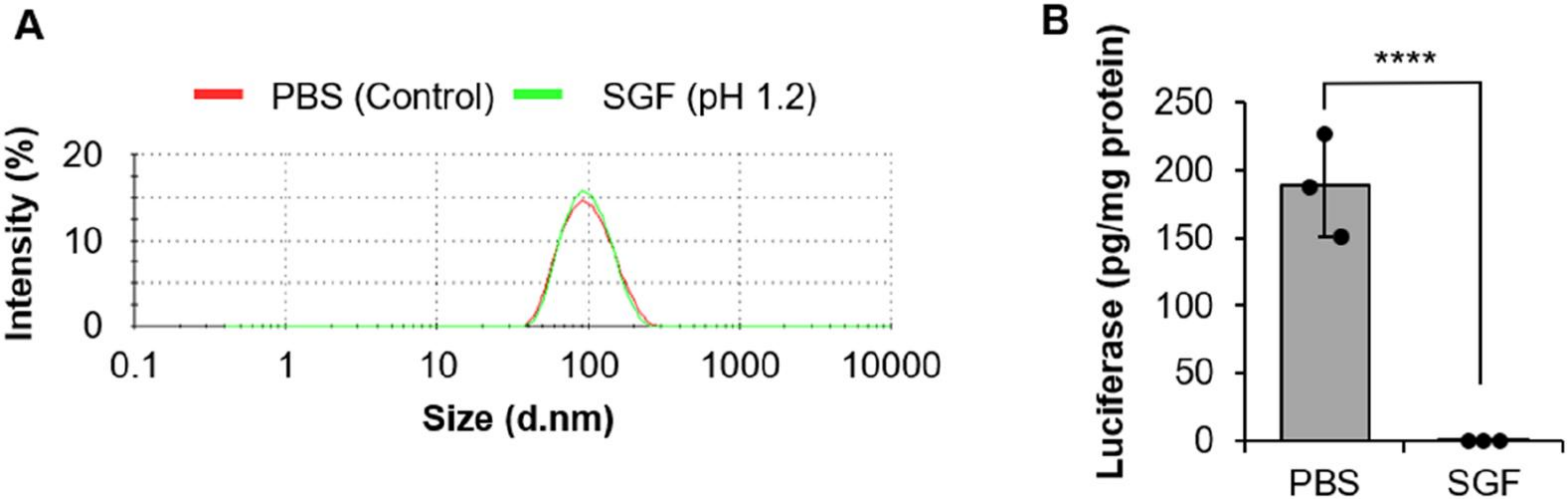
Characterization of SM‐102‐containing mRNA–LNPs treated with PBS (control) or SGF. (A) Particle size distribution of mRNA–LNP formulations. (B) Luciferase expression in Caco‐2 cells treated with mRNA–LNPs. Data are represented as mean ± standard deviation (SD; *n* = 3). ****p < 0.001 (student's *t*‐test).

The mRNA–LNPs exhibited typical physicochemical characteristics, with a particle size of 86.4 ± 3.5 nm, a polydispersity index (PDI) of 0.12 ± 0.011, and an EE of 97.7 ± 2.0%. SGF or PBS treatment did not significantly change particle size or EE compared with those of the untreated particles (Table 2, Figure 1A). In contrast, luciferase expression was significantly reduced by SGF treatment (Figure 1B), indicating that functionality of the mRNA–LNPs was impaired.

**Table 2:** Physicochemical properties of mRNA‐LNPs (SM‐102) treated with PBS (Control) and SGF (*n* = 3).

	Size (nm)	PDI	EE (%)
PBS (Control)	95.21 ± 3.21	0.140 ± 0.015	96.07 ± 2.76
SGF	95.21 ± 3.66	0.152 ± 0.052	94.47 ± 2.17

Abbreviations: EE, encapsulation efficiency; LNP, lipid nanoparticle; PBS, phosphate‐buffered saline; PDI, polydispersity index; SGF, simulated gastric fluid.

### 3.2 Effect of Pepsin on the mRNA–LNPs

In preliminary experiments, mRNA degradation was observed in SGF at pH 1.2. Therefore, to eliminate the confounding effect of mRNA degradation, subsequent experiments were conducted at pH 3, at which point the mRNA remained intact. Pepsin has been reported to retain its enzymatic activity even at pH 3 (Salelles et al. 2021).

**Figure 2:**
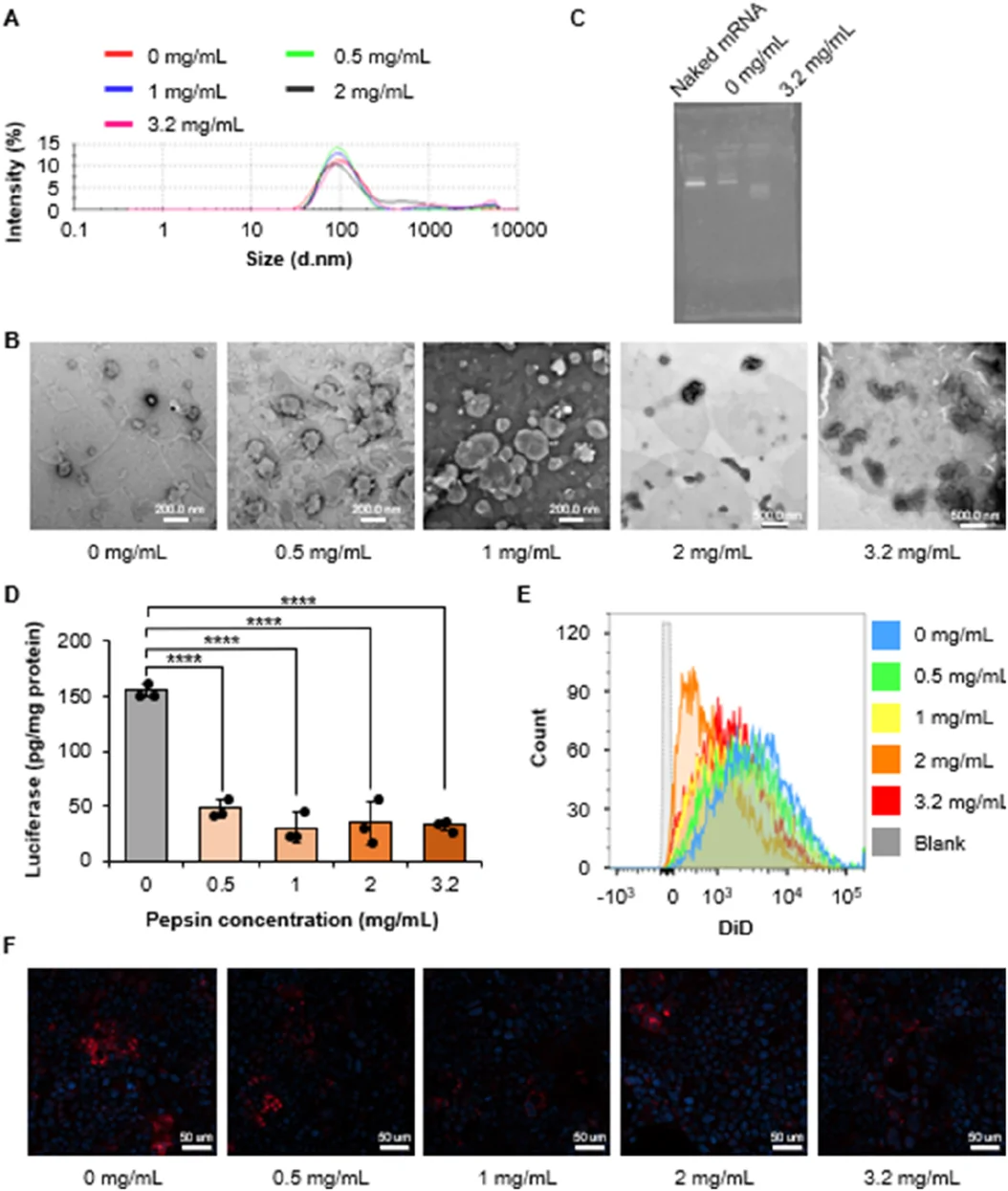
Characterization of SM‐102‐containing mRNA–LNPs treated with pepsin. (A) Particle size distribution of mRNA–LNP formulations. (B) Representative TEM images of the formulations. (C) Agarose gel electrophoresis of mRNA after treatment with pepsin (0, 3.2 mg/mL). (D) Luciferase expression in Caco‐2 cells treated with mRNA–LNPs. (E) Quantification of cellular uptake of mRNA–LNPs by flow cytometry. (F) CLSM images of Caco‐2 cells treated with mRNA–LNPs. DiD‐labeled mRNA–LNPs are shown in red, and nuclei stained with Hoechst 33342 are shown in blue. Data are represented as mean ± SD (*n* = 3). ****p < 0.001 (Dunnett's multiple comparison test).

Following pepsin treatment, a slight increase in particle size was observed with increasing pepsin concentration (Table 3, Figure 2A). PDI also increased consistently, indicating a deterioration in size uniformity. The enlargement of particle size and alterations in particle morphology were further confirmed by TEM (Figure 2B). To investigate the integrity of mRNA remaining within the particles after treatment, electrophoretic analysis was performed. In the group treated with pepsin at 0 mg/mL, a distinct mRNA band was observed, whereas in the group treated with 3.2 mg/mL pepsin, the band shifted toward the lower‐molecular‐weight region (Figure 2C). These findings suggest that exposure to pepsin induced degradation of the encapsulated mRNA. Because the electrophoretic band was markedly diminished following pepsin treatment, it is also possible that mRNA leakage from the particles occurred. Luciferase assay demonstrated that exposure to pepsin reduced the protein‐expression efficiency of the mRNA–LNPs (Figure 2D). In addition, flow cytometry and CLSM analyses revealed decreased cellular uptake of the particles following exposure to pepsin‐containing solutions (Figure 2E,F). Taken together, these results suggest that exposure to pepsin reduces the protein‐expression capability of mRNA–LNPs through both decreased cellular uptake caused by particle enlargement and degradation of encapsulated mRNA.

**Table 3:** Physicochemical properties of mRNA‐LNPs (SM‐102) treated with pepsin (*n* = 3).

Pepsin concentration (mg/mL)	Size (nm)	PDI
0	92.22 ± 3.57	0.197 ± 0.030
0.5	99.14 ± 9.66	0.185 ± 0.033
1	104.9 ± 4.8	0.227 ± 0.043
2	112.6 ± 8.1	0.286 ± 0.043
3.2	120.1 ± 7.3	0.317 ± 0.038

Abbreviations: LNP, lipid nanoparticle; PDI, polydispersity index

### 3.3 Effect of Bile Acids on the mRNA–LNPs

**Figure 3:**
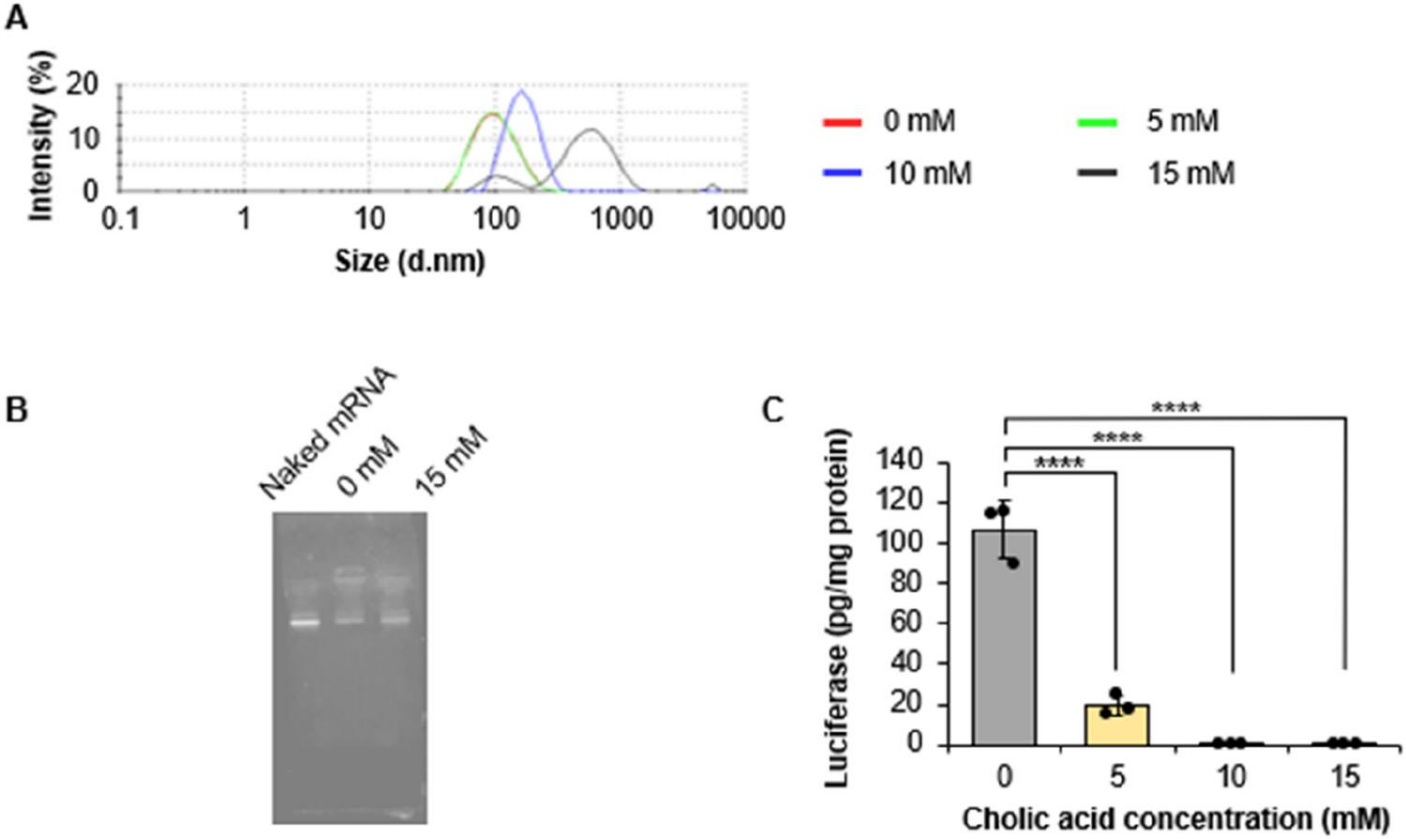
Characterization of SM‐102‐containing mRNA–LNPs treated with bile acid. (A) Particle size distribution of mRNA–LNP formulations. (B) Agarose gel electrophoresis of mRNA after treatment with cholic acid (0, 15 mM). (C) Luciferase expression in Caco‐2 cells treated with mRNA–LNPs. Data are represented as mean ± SD (*n* = 3). ****p < 0.001 (Dunnett's multiple comparison test).

Because bile acids possess surfactant properties, they may solubilize lipids of LNPs, thereby impairing particle function. Therefore, the effect of bile acid on the characteristics of mRNA*–*LNPs was evaluated. Particle size increased in a bile acid concentration‐dependent manner (Table 4, Figure 3A). Moreover, even low concentrations of bile acids resulted in a marked decrease in EE. Electrophoresis results demonstrated that exposure to bile acids did not affect the integrity of mRNA remaining within the particles after treatment (Figure 3B). Protein expression declined with the reduction in EE (Figure 3C). Bile acid‐induced dysfunction of mRNA*–*LNPs was probably attributable to mRNA leakage caused by lipid solubilization.

**Table 4:** Physicochemical properties of mRNA‐LNPs (SM‐102) treated with cholic acid (*n* = 3).

Cholic acid concentration (mM)	Size (nm)	PDI	EE (%)
0	93.11 ± 0.64	0.126 ± 0.018	97.99 ± 0.72
5	99.60 ± 6.95	0.169 ± 0.052	11.15 ± 2.27
10	156.2 ± 3.3	0.0762 ± 0.0152	7.53 ± 1.14
15	466.5 ± 16.3	0.495 ± 0.022	10.81 ± 1.04

Abbreviations: EE, encapsulation efficiency; LNP, lipid nanoparticle; PDI, polydispersity index.

### 3.4 Effect of Lipase on the mRNA–LNPs

**Figure 4:**
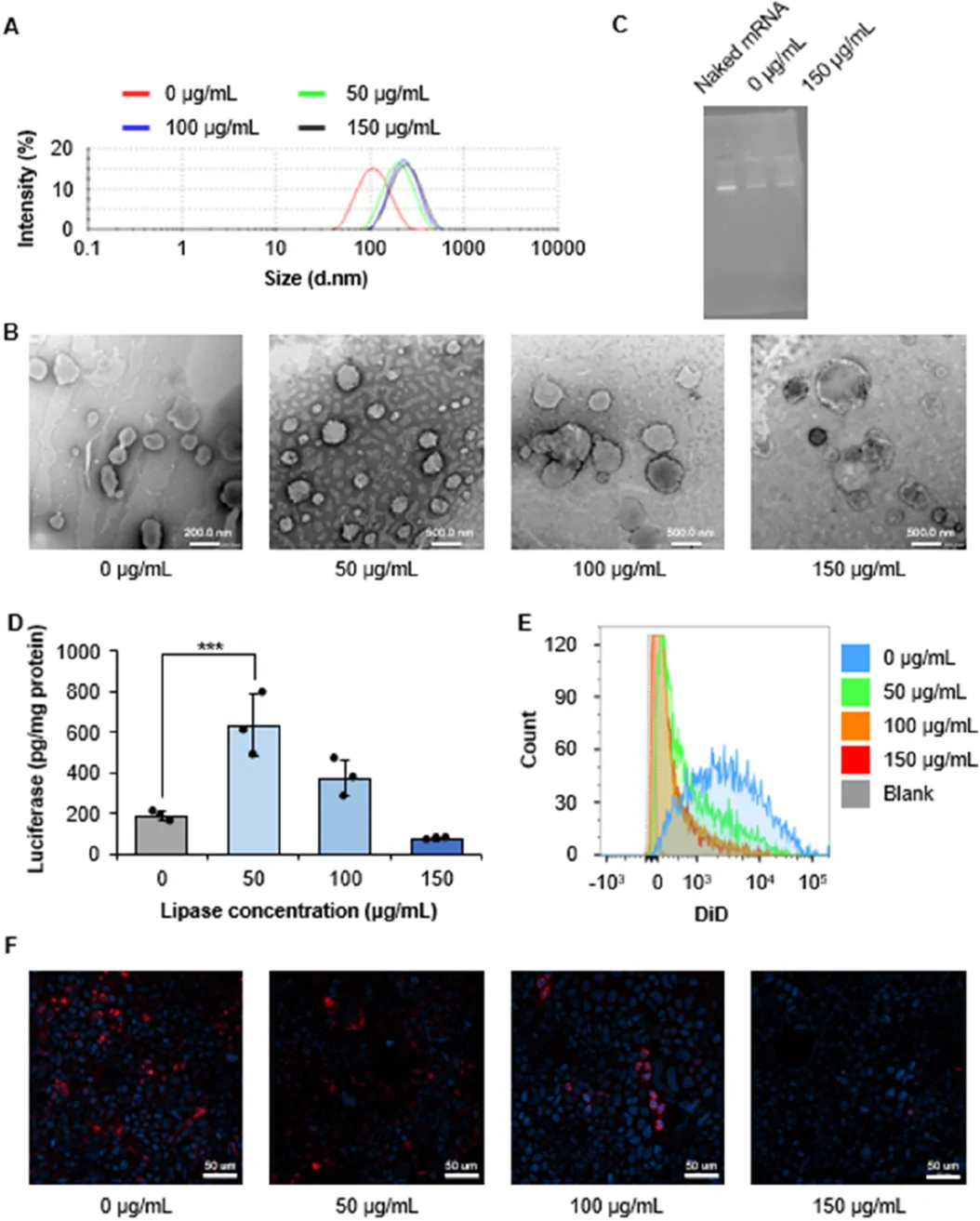
Characterization of SM‐102‐containing mRNA–LNPs treated with lipase. (A) Particle size distribution of mRNA–LNP formulations. (B) Representative TEM images of mRNA–LNP formulations. (C) Agarose gel electrophoresis of mRNA after treatment with lipase (0, 150 μg/mL). (D) Luciferase expression in Caco‐2 cells treated with mRNA–LNPs. (E) Quantification of cellular uptake of mRNA–LNPs by flow cytometry. (F) CLSM of Caco‐2 cells treated with mRNA–LNPs. DiD‐labeled mRNA‐LNPs are shown in red, and nuclei stained with Hoechst 33342 are shown in blue. Data are represented as mean ± SD (*n* = 3). ***p < 0.005 (Dunnett's multiple comparison test).

Lipase, which hydrolyzes ester bonds in lipids, may significantly impact structural integrity and functional properties of LNPs. Therefore, mRNA*–*LNPs were treated with various concentrations of lipase, and their characteristics were analyzed. Particle size increased with increasing lipase concentration (Table 5, Figure 4A), which was also confirmed by TEM (Figure 4B). EE decreased in a concentration‐dependent manner, suggesting that lipase destabilized the mRNA*–*LNP structure (Table 5). Electrophoresis results suggested that the integrity of mRNA encapsulated within the particles was maintained after exposure to lipase (Figure 4C). In contrast, protein expression did not show a strict concentration‐dependent relationship (Figure 4D). Notably, particles treated with low concentrations of lipase exhibited higher protein expression than that shown by the untreated group, whereas protein expression declined at relatively high lipase concentrations. Flow cytometry and CLSM revealed a concentration‐dependent decrease in intracellular uptake with increasing lipase concentrations (Figure 4E,F). Therefore, high concentrations of lipase impaired both the functionality and morphology of mRNA–LNPs, probably owing to hydrolysis of the ester bonds present in SM‐102 and DSPC, which compromised the functional properties of the constituent lipids.

**Table 5:** Physicochemical properties of mRNA‐LNPs (SM‐102) treated with lipase (*n* = 3).

Lipase concentration (μg/mL)	Size (nm)	PDI	EE (%)
0	95.29 ± 2.57	0.115 ± 0.013	94.78 ± 0.37
50	181.7 ± 3.8	0.125 ± 0.034	81.46 ± 1.72
100	205.1 ± 3.4	0.117 ± 0.009	70.48 ± 1.99
150	216.5 ± 5.7	0.135 ± 0.026	60.54 ± 1.92

Abbreviations: EE, encapsulation efficiency; LNP, lipid nanoparticle; PDI, polydispersity index.

### 3.5 Effect of Lipase on C12‐200‐containing mRNA–LNPs

We hypothesized that the use of lipids lacking ester bonds could mitigate lipase‐induced loss of mRNA*–*LNP functionality. Because the ionizable lipids are critical components of LNPs, we examined how the presence or absence of ester bonds within ionizable lipids influences mRNA*–*LNP functionality in the presence of lipase. Unlike SM‐102, C12‐200 does not contain ester bonds. Therefore, mRNA*–*LNPs were prepared using C12‐200 as the ionizable lipid and treated with lipase.

**Figure 5:**
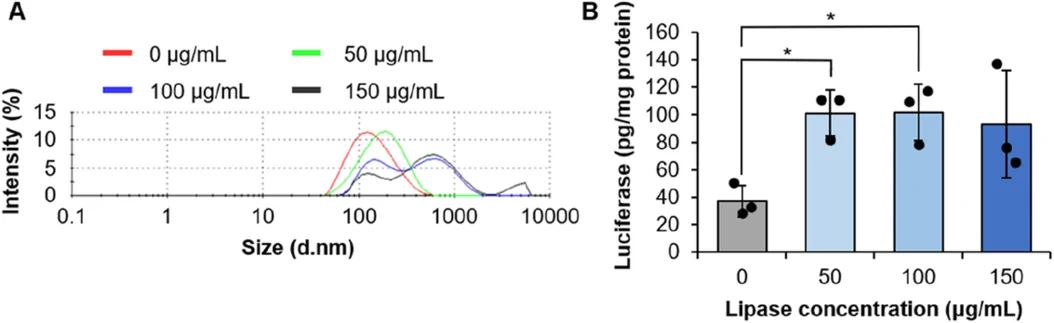
Characterization of C12‐200‐containing mRNA–LNPs treated with lipase. (A) Particle size distribution of C12‐200‐containing mRNA–LNP formulations. (B) Luciferase expression in Caco‐2 cells treated with C12‐200‐containing mRNA–LNPs. Data are represented as mean ± SD (*n* = 3). *p < 0.05 (Dunnett's multiple comparison test).

Untreated C12‐200‐containing mRNA*–*LNPs exhibited a particle size of 102.8 ± 1.6 nm, a PDI of 0.154 ± 0.008, and an EE of 47.05 ± 4.13%, which were consistent with previously reported characteristics of C12‐200‐based LNPs (Suri et al. 2025). Lipase treatment dose‐dependently increased particle size (Table 6, Figure 5A). However, lipase‐induced decrease in EE was attenuated compared to that observed for SM‐102‐containing mRNA*–*LNPs. Luciferase assay demonstrated no significant differences in protein expression among the lipase‐treated groups (Figure 5B). Collectively, these results suggested that reducing ester bonds within the particle formulation attenuated the detrimental effects of lipase on mRNA*–*LNP functionality.

**Table 6:** Physicochemical properties of mRNA‐LNPs (C12‐200) treated with lipase (*n* = 3).

Lipase concentration (μg/mL)	Size (nm)	PDI	EE (%)
Not treatment	102.8 ± 1.6	0.154 ± 0.008	47.05 ± 4.13
0	131.4 ± 20.2	0.230 ± 0.094	49.40 ± 5.02
50	156.9 ± 4.9	0.195 ± 0.027	43.99 ± 3.58
100	242.9 ± 19.2	0.375 ± 0.030	46.57 ± 2.02
150	335.7 ± 16.2	0.483 ± 0.013	32.34 ± 1.75

Abbreviations: EE, encapsulation efficiency; LNP, lipid nanoparticle; PDI, polydispersity index.

### 3.6 Effect of Amylase on the mRNA–LNPs

**Figure 6:**
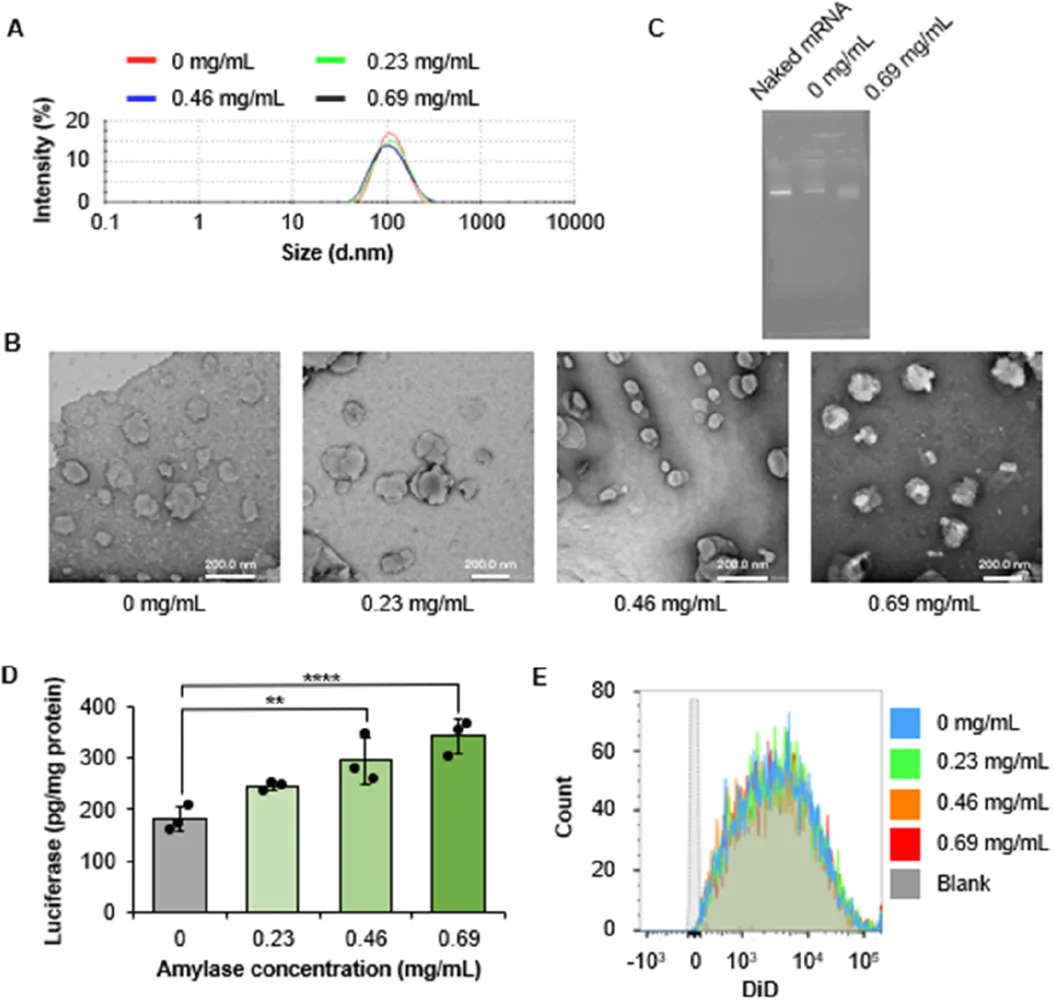
Characterization of SM‐102‐containing mRNA–LNPs treated with amylase. (A) Particle size distribution of mRNA–LNP formulations. (B) Representative TEM images of mRNA–LNP formulations. (C) Agarose gel electrophoresis of mRNA after treatment with amylase (0, 0.69 mg/mL). (D) Luciferase expression in Caco‐2 cells treated with mRNA–LNPs. (E) Quantification of cellular uptake of mRNA–LNPs by flow cytometry. Data are represented as mean ± SD (*n* = 3). **p < 0.01 and ****p < 0.001 (Dunnett's multiple comparison test).

Treatment with amylase, a pancreatic enzyme, did not affect particle size (Table 7, Figure 6A). TEM observations also revealed no notable changes in particle size or morphology (Figure 6B). In addition, EE showed no marked change; however, electrophoresis results suggested that a portion of the encapsulated mRNA may have undergone degradation (Figure 6C). Because neither the particle morphology nor encapsulation efficiency was significantly altered, the degraded mRNA was likely localized near the particle surface. Taken together, these findings suggest that amylase does not markedly affect the physicochemical properties of mRNA–LNPs, although it may promote partial degradation of mRNA. Interestingly, protein expression increased with an increase in amylase concentration (Figure 6D). Flow cytometry revealed no substantial differences in cellular uptake after amylase treatment (Figure 6E). Collectively, these results indicated that amylase did not exert detrimental effects on the function of mRNA–LNPs. Because the degradation of encapsulated mRNA appeared to be partial and limited, it may not have been sufficient to reduce protein expression activity.

**Table 7:** Physicochemical properties of mRNA‐LNPs (SM‐102) treated with amylase (*n* = 3).

Amylase concentration (mg/mL)	Size (nm)	PDI	EE (%)
0	100.3 ± 0.6	0.160 ± 0.040	99.44 ± 0.20
0.23	98.24 ± 3.72	0.140 ± 0.011	97.58 ± 0.68
0.46	95.39 ± 2.21	0.127 ± 0.016	98.42 ± 0.44
0.69	95.59 ± 2.65	0.136 ± 0.007	96.67 ± 2.41

Abbreviations: EE, encapsulation efficiency; LNP, lipid nanoparticle; PDI, polydispersity index.

### 3.7 Effects of Digestive Enzymes on ALC‐0315‐Based mRNA–LNPs

To evaluate the relationship between lipid composition and susceptibility to digestive enzymes, experiments similar to those described above were performed using mRNA–LNPs formulated with ALC‐0315 as the ionizable lipid. Prior to treatment, the ALC‐0315‐based mRNA–LNPs exhibited a particle size of 102.9 ± 1.0 nm, a PDI of 0.172 ± 0.072, and EE of 97.91 ± 0.60%.

**Figure 7:**
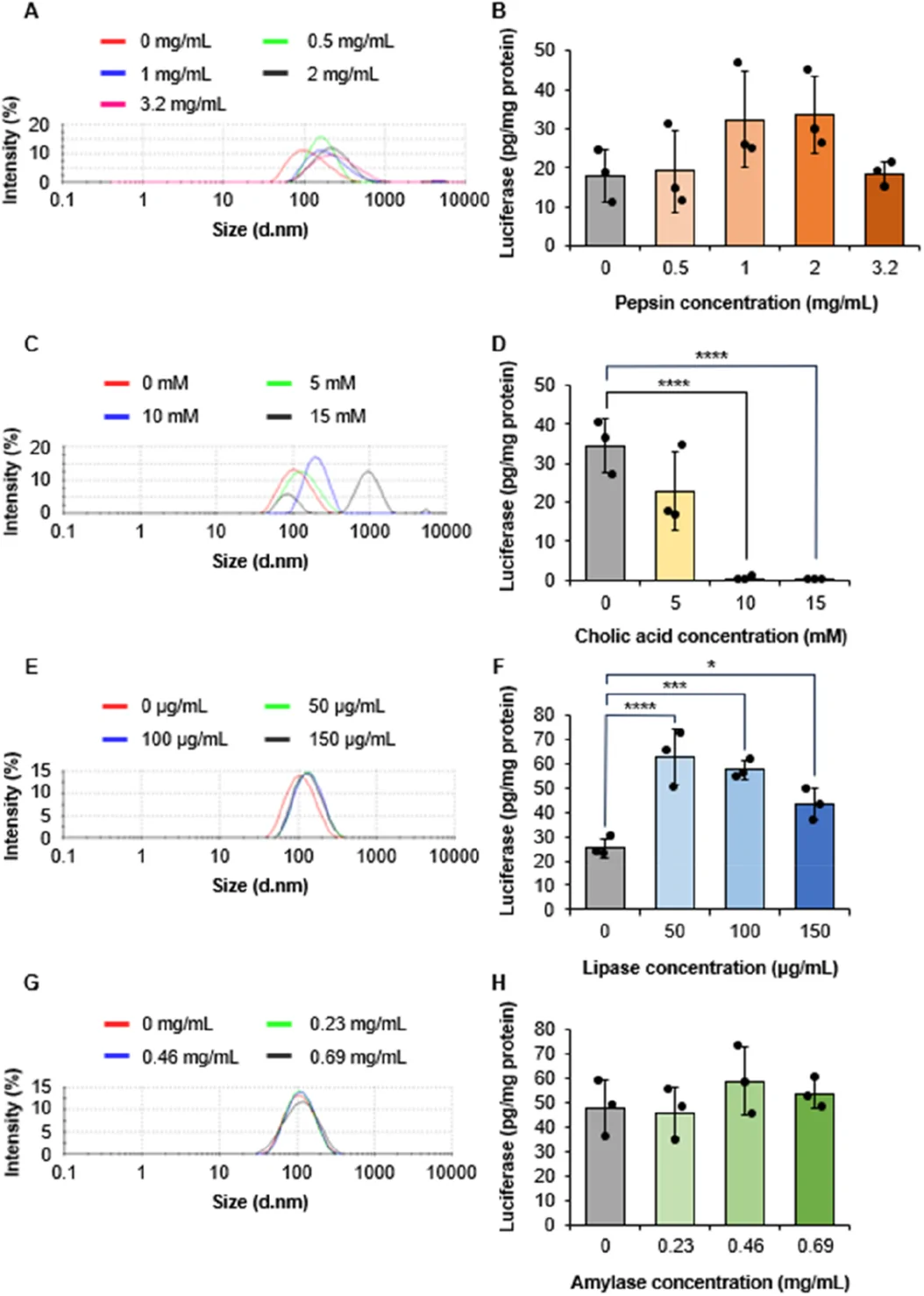
Characterization of ALC‐0315‐containing mRNA–LNPs treated with each enzyme. (A) Particle size distribution of mRNA–LNPs treated with pepsin. (B) Luciferase expression in Caco‐2 cells added with mRNA–LNPs treated with pepsin. (C) Particle size distribution of mRNA–LNPs treated with cholic acid. (D) Luciferase expression in Caco‐2 cells added with mRNA–LNPs treated with cholic acid. (E) Particle size distribution of mRNA–LNPs treated with lipase. (F) Luciferase expression in Caco‐2 cells added with mRNA–LNPs treated with lipase. (G) Particle size distribution of mRNA–LNPs treated with amylase. (H) Luciferase expression in Caco‐2 cells added with mRNA–LNPs treated with amylase. Data are represented as mean ± SD (*n* = 3). *p < 0.05, **p < 0.01 and ****p < 0.001 (Dunnett's multiple comparison test).

Following exposure to pepsin, particle size increased, suggesting particle aggregation, as was also observed for SM‐102‐based mRNA–LNPs (Table 8, Figure 7A). However, unlike SM‐102‐based particles, no significant reduction in protein expression was observed compared with the untreated group (Figure 7B).

**Table 8:** Physicochemical properties of mRNA‐LNPs (ALC‐0315) treated with pepsin (*n* = 3).

Pepsin concentration (mg/mL)	Size (nm)	PDI
0	108.9 ± 1.0	0.216 ± 0.011
0.5	167.0 ± 20.9	0.153 ± 0.009
1	173.5 ± 13.4	0.190 ± 0.018
2	197.6 ± 4.6	0.234 ± 0.039
3.2	204.3 ± 9.1	0.240 ± 0.019

Abbreviations: LNP, lipid nanoparticle; PDI, polydispersity index.

Following exposure to cholic acids, particle size increased markedly, and EE decreased substantially (Table 9, Figure 7C). In addition, luciferase expression was significantly reduced (Figure 7D). These changes were consistent with those observed for SM‐102‐based mRNA–LNPs, suggesting that the susceptibility of mRNA–LNPs to surfactant‐mediated damage is largely independent of the ionizable lipid used.

**Table 9:** Physicochemical properties of mRNA‐LNPs (ALC‐0315) treated with cholic acid (*n* = 3).

Cholic acid concentration (mM)	Size (nm)	PDI	EE (%)
0	101.5 ± 1.7	0.158 ± 0.007	98.25 ± 0.41
5	121.3 ± 0.9	0.176 ± 0.022	7.21 ± 2.81
10	188.0 ± 2.1	0.103 ± 0.017	5.30 ± 2.42
15	436.6 ± 77.7	0.956 ± 0.069	8.55 ± 3.15

Abbreviations: EE, encapsulation efficiency; LNP, lipid nanoparticle; PDI, polydispersity index.

In contrast to SM‐102‐based particles, ALC‐0315‐based mRNA–LNPs did not exhibit a notable increase in particle size following lipase treatment (Figure 7E). However, a decrease in EE was observed (Table 10). Because ALC‐0315 also contains ester bonds, it is likely susceptible to lipase‐mediated hydrolysis. Furthermore, the profile of protein expression following lipase treatment was similar to that observed for SM‐102‐based particles, with lower lipase concentrations resulting in enhanced protein expression and higher concentrations causing functional impairment (Figure 7F). These findings further support the notion that low levels of lipase may enhance the protein‐expression capability of mRNA–LNPs.

**Table 10:** Physicochemical properties of mRNA‐LNPs (ALC‐0315) treated with lipase (*n* = 3).

Lipase concentration (μg/mL)	Size (nm)	PDI	EE (%)
0	109.6 ± 14.0	0.202 ± 0.067	98.84 ± 0.59
50	131.8 ± 3.6	0.143 ± 0.020	41.20 ± 2.42
100	126.1 ± 1.0	0.133 ± 0.005	40.22 ± 2.98
150	122.7 ± 2.0	0.124 ± 0.005	40.94 ± 3.37

Abbreviations: EE, encapsulation efficiency; LNP, lipid nanoparticle; PDI, polydispersity index.

Following exposure to amylase, neither particle size nor EE changed substantially, which was consistent with the results obtained for SM‐102‐based mRNA–LNPs (Table 11, Figure 7G). However, unlike SM‐102‐based particles, protein expression did not increase with increasing amylase concentration (Figure 7H). This finding suggests that the effect of amylase on protein expression may depend on the lipid composition of the mRNA–LNPs.

**Table 11:** Physicochemical properties of mRNA‐LNPs (ALC‐0315) treated with amylase (n = 3).

Amylase concentration (mg/mL)	Size (nm)	PDI	EE (%)
0	100.6 ± 2.4	0.154 ± 0.027	98.43 ± 0.24
0.23	102.7 ± 0.8	0.143 ± 0.010	96.86 ± 0.17
0.46	102.7 ± 1.4	0.168 ± 0.019	96.83 ± 0.87
0.69	101.7 ± 0.7	0.158 ± 0.004	95.38 ± 1.13

Abbreviations: EE, encapsulation efficiency; LNP, lipid nanoparticle; PDI, polydispersity index.

### 3.8 Effect of pH on the mRNA–LNPs

As shown in Figure 1B, treatment with simulated gastric fluid (SGF) at pH 1.2 resulted in a marked loss of mRNA‐LNP functional activity. To further investigate the effects of acidic conditions on the mRNA–LNPs, we evaluated their physicochemical properties and protein‐expression efficiency after treatment with buffer solutions of various pH. Differentiated Caco‐2 cells form tight junctions and a mucus layer; therefore, HEK293 cells were used to directly assess intrinsic protein expression.

**Figure 8:**
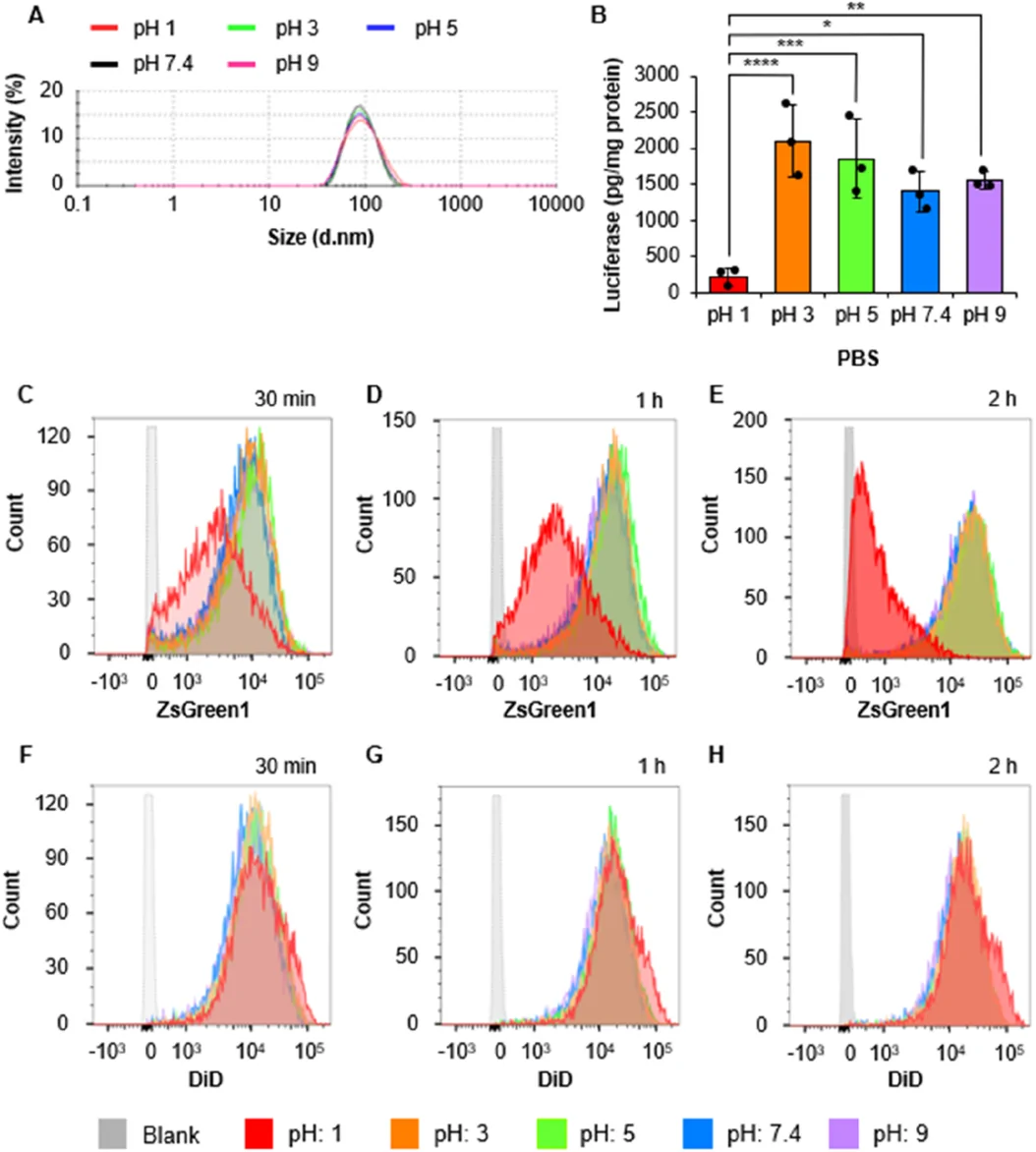
Characterization of SM‐102‐containing mRNA–LNPs treated with pH‐adjusted PBS. (A) Particle size distribution of mRNA–LNP formulations. (B) Luciferase expression in Caco‐2 cells treated with mRNA–LNPs. (C–E) Flow cytometric analysis of ZsGreen1 expression in Caco‐2 cells treated with mRNA‐LNPs after PBS treatment for (C) 30 min, (D) 1 h, and (E) 2 h. (F–H) Quantification of cellular uptake of mRNA–LNPs by flow cytometry after PBS treatment for (F) 30 min, (G) 1 h, and (H) 2 h. Data are represented as mean ± SD (n = 3). *p < 0.05, **p < 0.01, ***p < 0.005 and ****p < 0.001 (Tukey's multiple comparison test).

Changes in pH did not significantly affect particle size or EE (Table 8, Figure 8A). In contrast, protein expression was markedly reduced in cells treated with mRNA–LNPs and exposed to PBS of pH 1 (Figure 8B), consistent with the results observed following SGF treatment. No reduction in protein expression was observed in cells treated with particles exposed to PBS pH ≥ 3. These findings indicated that functional impairment of mRNA–LNPs under acidic conditions occurred only at extremely low pH.

Flow cytometry was performed to further evaluate the cellular uptake of mRNA–LNPs treated with PBS of various pH. To simultaneously assess protein expression and intracellular uptake, fluorescence derived from expressed ZsGreen1 and DiD‐labeled LNPs was quantified. A significant reduction in ZsGreen1 expression was observed exclusively in the group treated with PBS of pH 1, which was consistent with the results of luciferase assay (Figure 8C). However, no notable changes in protein expression were observed in groups treated with PBS of pH ≥ 3 when the treatment duration was extended, whereas the group treated with PBS of pH 1 exhibited a further decrease in protein expression (Figure 8D, E). These results suggested that prolonged exposure to extremely low pH exacerbated the loss of mRNA–LNP functionality. In contrast, intracellular uptake across different pH conditions revealed no significant differences among the groups, regardless of treatment duration (Figure 8F–H).

### 3.9 Effect of pH on mRNA Integrity

**Figure 9:**
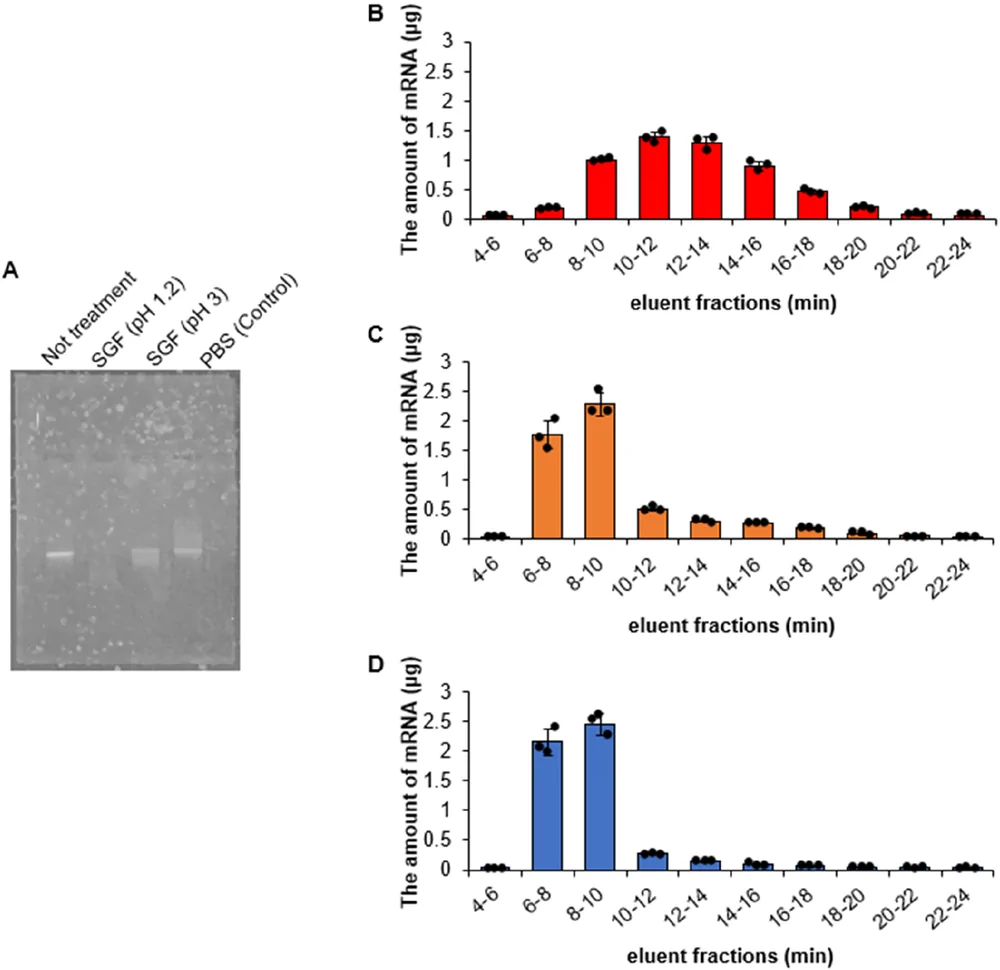
Evaluation of mRNA integrity after exposure to SGF or PBS. (A) Agarose gel electrophoresis of mRNA after treatment with SGF (pH 1.2), SGF (pH 3), or PBS (control). GPC analysis of mRNA after treatment with (B) SGF (pH 1.2), (C) SGF (pH 3), and (D) PBS. Data are represented as mean ± SD (*n* = 3).

Cellular uptake of mRNA–LNPs was not affected by strongly acidic solutions; therefore, we hypothesized that the mRNA itself might be inactivated under such conditions, and examined the effect of acidity on structural integrity of mRNA. In the group treated with SGF at pH 1.2, the mRNA band was not detectable, indicating extensive degradation. In contrast, the mRNA band was clearly visible in the group treated with SGF at pH 3 (Figure 9A). In GPC, a delayed elution profile was observed for the SGF (pH 1.2)‐treated group, suggesting structural breakdown or fragmentation of mRNA (Figure 9B). In contrast, no such delay was observed in the SGF (pH 3)‐ or PBS‐treated group (Figure 9C,D), indicating that the loss of protein‐expression efficiency of mRNA–LNPs exposed to SGF was attributable to degradation of the encapsulated mRNA under highly acidic conditions.

## 4 Discussion

In this study, we systematically evaluated the effects of major GI components on the functionality of mRNA–LNPs to assess their suitability for oral administration. Although several alternative carrier systems for oral RNA delivery, such as exosomes, have been reported (Pomatto et al. 2023), these platforms lack clinical precedents and may present regulatory and safety‐related challenges. In contrast, mRNA–LNPs have been widely used in clinical settings, making them an attractive and pragmatic platform for translational research. SGF, pepsin, bile acids, and lipase at high concentrations significantly reduced the protein‐expression efficiency of mRNA–LNPs. Collectively, these findings provide important insights into the key GI factors that limit the performance of oral mRNA–LNPs and offer a rational basis for designing highly robust strategies for oral delivery of mRNA–LNPs.

The detrimental effect of SGF was primarily attributed to its extremely acidic condition. However, the functionality of mRNA–LNPs was not affected at pH ≥ 3, suggesting that increasing gastric pH to ≥ 3 prior to mRNA–LNP administration may represent an effective strategy to mitigate the adverse effects of gastric acid. Furthermore, degradation of encapsulated mRNA was a major contributor to the observed loss of function. Therefore, preventing direct exposure of encapsulated mRNA to the external acidic environment may be a viable approach for protecting mRNA–LNPs against gastric acid‐induced degradation.

Pepsin and bile acids significantly reduced the functionality of mRNA–LNPs. These observations were consistent with those of previous studies. Exposure to bile acids and pepsin reduce the gene‐knockdown efficiency of small interfering RNA (siRNA)‐loaded lipid nanoparticles (Ball et al. 2018). Although the type and biological function of encapsulated RNA differ between siRNA and mRNA, the concordant trends observed in both studies suggest that these GI components primarily exert detrimental effects through interactions with the lipid constituents of nanoparticles, rather than directly degrading the RNA cargo. Therefore, preventing direct contact between mRNA–LNPs and GI substances, such as pepsin and bile acids, is considered an effective strategy for preserving nanoparticle functionality. One potential approach involves coating particles with a protective polymer layer that can inhibit interactions between the lipid components and external agents. Supporting this concept, liposomes have been successfully protected from bile acid‐induced destabilization by coating with chitosan and the enteric polymer Eudragit S100 (Barea et al. 2012). Therefore, polymer‐based surface modification is a promising and feasible strategy for enhancing the GI stability of mRNA–LNPs.

Particles treated with low concentrations of lipase exhibited enhanced protein expression, whereas those treated with high concentrations showed markedly reduced functionality. Given that lipase treatment reduced cellular uptake of mRNA–LNPs, enhanced protein expression observed at low lipase concentrations was likely attributable to factors other than increased uptake. One possible explanation is partial lipid remodeling, which transiently improves endosomal escape or intracellular release of mRNA, although this mechanism requires further investigation. Furthermore, reducing the number of ester bonds in the lipid components attenuated the effects of lipase, indicating that mRNA–LNPs were susceptible to lipase‐mediated hydrolysis. Although C12‐200‐based mRNA–LNPs have been previously explored for oral delivery applications (Suri et al. 2025), their susceptibility to lipase has not been reported. Therefore, our findings provide new evidence that avoiding ester bonds in ionizable lipids is a rational and effective strategy for mitigating lipase‐mediated degradation and preserving functional performance of mRNA–LNPs in the GI environment.

In the present study, SGF exposure degraded the mRNA of mRNA–LNPs. RNA undergoes hydrolysis under strongly acidic and high‐temperature conditions (Jarvinen et al. 1991). Consistent with this report, our results suggest that mRNA exposed to artificial gastric fluid is susceptible to acid‐induced hydrolysis. Notably, no degradation was observed in solutions of pH 3, indicating that this process was highly pH‐dependent. With regard to degradation of the lipid components following exposure to digestive enzymes, our findings suggest that lipase may induce hydrolysis of ester bonds present in the constituent lipids of mRNA–LNPs. In contrast, the other digestive enzymes examined in this study are not known to utilize lipids as substrates and are therefore unlikely to directly contribute to lipid degradation. Nevertheless, the present study did not directly characterize the chemical structures of lipids after enzymatic treatment. Accordingly, a detailed investigation of lipid structural changes following exposure to digestive enzymes remains an important subject for future research.

The relationship between lipid composition and nanoparticle stability has been extensively investigated. DSPC, a phospholipid component of mRNA–LNPs, is believed to contribute to particle stabilization owing to its cylindrical molecular geometry. In addition, cholesterol has been reported to enhance nanoparticle stability by regulating membrane integrity and rigidity (Hou et al. 2021). Previous studies have also demonstrated that optimization of lipid composition can improve the gastrointestinal stability of liposomes, another class of lipid‐based nanoparticles (Wu et al. 2015). Although these findings provide useful insights into the design of orally administered mRNA–LNPs, liposomes and mRNA–LNPs differ substantially in their internal structures and physicochemical properties. Therefore, the functions of individual lipid components may not be directly comparable between the two systems. Consequently, studies specifically focused on mRNA–LNPs, such as the present work, are necessary to better understand the factors governing their stability within the gastrointestinal tract.

In the present study, we investigated the relationship between ionizable lipid composition and gastrointestinal stability by comparing mRNA–LNPs formulated with different ionizable lipids. With respect to the other lipid components, DSPC may also contribute to the susceptibility of mRNA–LNPs to lipase‐mediated degradation. Because DSPC contains ester bonds within its molecular structure, it may undergo hydrolysis in a manner similar to that of ester‐containing ionizable lipids. Consistent with this possibility, phospholipids incorporated into liposomes have previously been reported to be degraded by lipase (Wu et al. 2015). In contrast, DMG‐PEG2000 is designed to dissociate from the particle surface after administration and is therefore unlikely to make a substantial contribution to gastrointestinal stability. These observations suggest that, in addition to the ionizable lipid, other lipid components may influence the stability of mRNA–LNPs in the gastrointestinal environment and should be considered when designing formulations for oral delivery.

This study provides important insights for developing mRNA–LNPs for oral administration. Delivering mRNA–LNPs via the oral route also opens new therapeutic possibilities for diseases localized within the GI tract. One such target is colorectal cancer, which is the third most common cancer worldwide and a leading cause of cancer‐related mortality (Mármol et al. 2017). Oral administration of therapeutic mRNA may enable localized treatment at the disease site, potentially minimizing systemic adverse effects. Research on mRNA‐based cancer therapies is rapidly advancing, and several candidates have entered clinical trials (Hou et al. 2021), supporting the feasibility of applying them against colorectal cancer via oral delivery. Additionally, orally administered mRNA formulations may be applicable as vaccines against GI infections. Norovirus, for example, infects the intestinal mucosa (Robilotti et al. 2015). While parenteral vaccines primarily induce systemic immunity, oral vaccines can stimulate mucosal immune responses in the gut, potentially leading to relatively highly effective protective immunity (Kirtane et al. 2023). Furthermore, enhanced uptake of mRNA–LNPs by Caco‐2 cells, which serve as a model of the GI epithelium, has been demonstrated (Shinkai et al. 2024). Consistent with this concept, mRNA‐based vaccines against norovirus are currently under clinical development (Atochina‐Vasserman et al. 2024), supporting the potential feasibility of orally administrable mRNA‐based norovirus vaccines. However, this study does not address specific particle design strategies for orally administered mRNA–LNPs. Therefore, further investigations based on the findings of this study will be necessary to achieve the practical realization of oral mRNA–LNP delivery.

## 5 Conclusions

This study was conducted as an initial step toward the development of orally administrable mRNA–LNPs. To our knowledge, this is the first report to systematically investigate the effects of individual gastrointestinal components on the functionality of mRNA–LNPs. The findings of the present study identify the key barriers to oral mRNA–LNP delivery and provide a rational basis for developing protective strategies to mitigate destabilization of mRNA–LNPs in the GI tract. However, future studies should assess the effectivity and safety of such formulations *in vivo* to ensure their clinical applicability.

## Supporting information


**Figure S1:** IVIS analysis of luciferase expression following oral administration of SM‐102‐based mRNA–LNPs in mice. Representative IVIS images obtained 6 h after oral administration of naked mRNA showing (A) whole‐body, (B) liver, and (C) gastrointestinal tract signals. Representative IVIS images obtained 6 h after oral administration of mRNA–LNPs showing (D) whole‐body, (E) liver, and (F) gastrointestinal tract signals.

## Data Availability

The data that support the findings of this study are available from the corresponding author upon reasonable request.
