# Supplementary material for: Comprehensive Evaluation of Gastrointestinal Factors Influencing the Stability and Function of mRNA‐Encapsulated Lipid Nanoparticles
Source: Drug Dev Res. 2026 Sep 23;87(7):e70392. doi: 10.1002/ddr.70392 (PMC13598902; doi:10.1002/ddr.70392)
Supplement: Supplementary file 1 [file DDR-87-e70392-s001.docx]

**Supplementary material**

Comprehensive Evaluation of Gastrointestinal Factors Influencing the Stability and Function of mRNA-encapsulated Lipid Nanoparticles

Toma Shinkai^1^, Koki Ogawa^1^*, Mohamed S. Mohamed ^1,2^, Tetsuya Ozeki^1^*

1) Drug Delivery and Nano Pharmaceutics, Graduate School of Pharmaceutical Sciences, Nagoya City University, 3-1 Tanabe-dori, Mizuho-ku, Nagoya, Aichi 467-8603, Japan
2) Department of Pharmaceutics and Pharmaceutical Technology, Faculty of Pharmacy, Al-Azhar University, Assiut Branch, Assiut 71524, Egypt

***Corresponding author**

Koki Ogawa

Drug Delivery and Nano Pharmaceutics, Graduate School of Pharmaceutical Sciences, Nagoya City University, 3-1 Tanabe-dori, Mizuho-ku, Nagoya, Aichi 467-8603, Japan

E-mail: kogawa@phar.nagoya-cu.ac.jp

ORCID: 0000-0002-6248-0690

Tetsuya Ozeki

Drug Delivery and Nano Pharmaceutics, Graduate School of Pharmaceutical Sciences, Nagoya City University, 3-1 Tanabe-dori, Mizuho-ku, Nagoya, Aichi 467-8603, Japan

E-mail: ozekit@phar.nagoya-cu.ac.jp

ORCID: 0000-0002-4488-2127

**Figure S1.** IVIS analysis of luciferase expression following oral administration of SM-102-based mRNA–LNPs in mice. Representative IVIS images obtained 6 h after oral administration of naked mRNA showing (A) whole-body, (B) liver, and (C) gastrointestinal tract signals. Representative IVIS images obtained 6 h after oral administration of mRNA–LNPs showing (D) whole-body, (E) liver, and (F) gastrointestinal tract signals.
